# Biochemical Characterization and Synthetic Application of WciN and Its Mutants From *Streptococcus pneumoniae* Serotype 6B

**DOI:** 10.3389/fchem.2022.914698

**Published:** 2022-06-15

**Authors:** Wei Gong, Min Liang, Jielin Zhao, Hong Wang, Zonggang Chen, Fengshan Wang, Guofeng Gu

**Affiliations:** ^1^ National Glycoengineering Research Center, Shandong Provincial Key Laboratory of Carbohydrate Chemistry and Glycobiology, Shandong University, Qingdao, China; ^2^ School of Pharmaceutical Science, Shandong University, Jinan, China; ^3^ NMPA Key Laboratory for Quality Research and Evaluation of Carbohydrate-based Medicine, Shandong University, Qingdao, China

**Keywords:** *Streptococcus pneumoniae* serotype 6B, capsular polysaccharides, galactosyltransferase, site mutation, enzymatic synthesis

## Abstract

The biochemical properties of α-1,3-galactosyltransferase WciN from *Streptococcus pneumoniae* serotype 6B were systemically characterized with the chemically synthesized Glcα-PP-(CH_2_)_11_-OPh as an acceptor substrate. The *in vitro* site-directed mutation of D38 and A150 residues of WciN was further investigated, and the enzymatic activities of those WciN mutants revealed that A150 residue was the pivotal residue responsible for nucleotide donor recognition and the single-site mutation could completely cause pneumococcus serotype switch. Using WciN_A150P_ and WciN_A150D_ mutants as useful tool enzymes, the disaccharides Galα1,3Glcα-PP-(CH_2_)_11_-OPh and Glcα1,3Glcα-PP-(CH_2_)_11_-OPh were successfully prepared in multi-milligram scale in high yields.

## Introduction

Pneumonia caused by the Gram-positive pathogen *Streptococcus pneumoniae* is a highly fatal infectious disease worldwide. The extracellular capsular polysaccharides (CPSs) abundantly coating the pneumococcal cell surfaces are recognized as one of the predominant causative virulence factors owing to their enhancing resistance to the complement-mediated opsonophagocytosis (Alonsodevelasco et al., 1995; Neeleman et al., 1999). Furthermore, these CPSs are identified as effective antigenic epitopes for the development of pneumococcal vaccines because of their inducing serotype-specific immunoprotection (Alonsodevelasco et al., 1995). Based on the confirmed serological profiles and unique CPS structures, more than 100 individual pneumococcal serotypes have been characterized and identified thus far (Henrichsen, 1995; Ganaie et al., 2020; Pimenta et al., 2021). Such a large diversity of CPS among pneumococcal serotypes has made it a huge challenge in CPS-related pneumococcal vaccine development.

**FIGURE 1:**
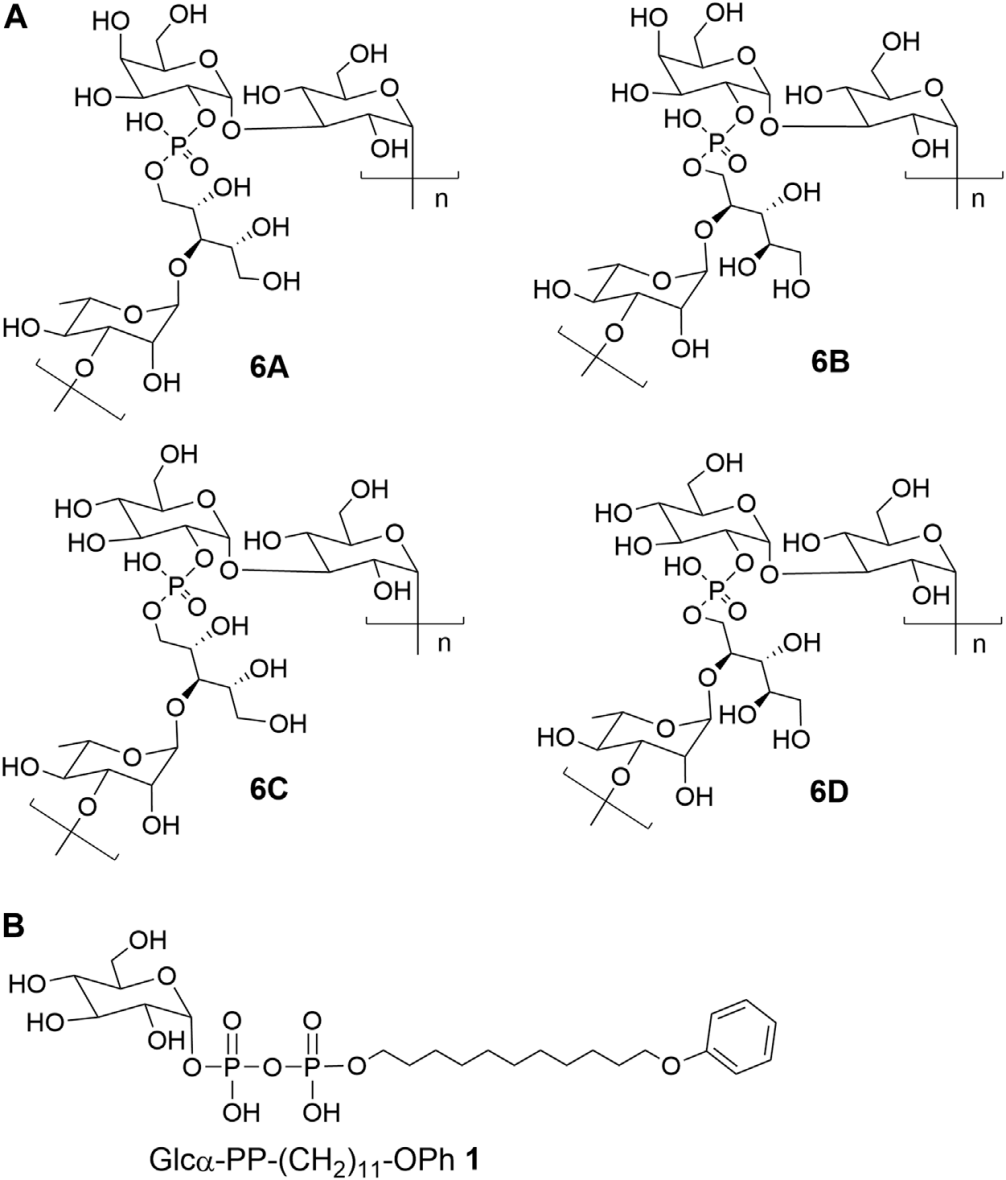
The chemical structures of **(A)** CPS repeat units of pneumococcal serotypes **6A–D** and **(B)** the glycolipid Glcα-PP-(CH_2_)_11_-OPh **1**.

Each capsular polysaccharide is programmatically synthesized by a series of enzymes encoded by the *cps* locus genes (Muñoz et al., 1997). It has been disclosed that the generation of antigenic diversity of CPS mainly contributed to elevated recombination and substitution rates of the *cps* locus (Mostowy et al., 2017). Although recombination within the *cps* locus has been assumed as the underlying cause for serotype evolution (Joshi et al., 2020), several studies revealed that serotype switching in pneumococcus was also implicated with the site-mutation of glycosyltransferase genes located in the *cps* locus (van Selm et al., 2003; Mavroidi et al., 2004; Mavroidi et al., 2007; Sheppard et al., 2010; Oliver et al., 2013). Serogroup six of *S. pneumoniae* has been characterized to contain eight serotypes including 6A-H (Park et al., 2015; van Tonder et al., 2015). Among them, the identified chemical structures of CPS 6A-D were shown in Figure 1A. The serotypes 6A and 6B have identical structures of capsular polysaccharides repeat unit (RU) only with a difference in the rhamnosidic linkage, which is α-1,3 linkage in 6A and α-1,4 linkage in 6B (Rebers and Heidelberger, 1961). Further studies revealed that the substitution of the catalytic triad residues, Ala192-Ser195-Arg254, in rhamnosyltransferase (WciP) could completely cause a serotype switch between 6A and 6B (Mavroidi et al., 2004; Sheppard et al., 2010). The CPS RUs of serotypes 6C and 6D have the glucose residue in place of the galactose residue in serotypes 6A and 6B through entire gene replacement of glycosyltransferase WciN, which has been respectively recognized as galactosyltransferase in 6A and 6B and as glucosyltransferase in 6C and 6D (Park et al., 2007a; Park et al., 2007b; Jin et al., 2009; Bratcher et al., 2010). Nevertheless, it has been disclosed that mutagenesis of A150 and/or D38 residues of WciN in serotype 6A or 6B resulted in a novel hybrid serotype 6F or 6G, which was identified as a different mixture ratio of 6A/6C for 6F, or 6B/6D for 6G, respectively (Oliver et al., 2013).

As mentioned above, WciN from pneumococcus type 6B strain is presumed as α-1,3-galactosyltransferase responsible for the RU assembly in the biosynthesis of type 6B CPS. Its galactosylation activity has been preliminarily investigated and identified *in vitro* using a chemosynthetic Und-PP-Glc surrogate, that is, Glcα-PP-(CH_2_)_10_CH_3_, as an acceptor substrate (Han et al., 2012). However, its detailed biochemical properties have not been reported yet. Moreover, the *in vivo* allelic exchange study has disclosed that the mutagenesis of aspartic acid residue at position 38 and alanine residue at position 150 of WciN could trigger pneumococcus serotype switch. Thus, the more *in vitro* evidence to confirm such serotype evolution are further worthy of exploring. In this study, we presented in detail the biochemical properties of WciN derived from pneumococcus type 6B using the synthesized glycolipid Glcα-PP-(CH_2_)_11_-OPh **1** (Wang et al., 2019; Wang et al., 2021; Liang et al., 2022) (Figure 1B) as acceptor substrate, and carried out the single or dual amino acid substitution of D38 and/or A150 residue of WciN to verify the different glycosylation functions of the resultant glycosyltransferase mutants.

## Materials and Methods

### Materials

Sugar nucleotide donors UDP-Gal and UDP-Glc were prepared as previously described (Li et al., 2020). Enzymatic acceptor substrate Glcα-PP-(CH_2_)_11_-OPh **1** was synthesized followed the protocol reported previously (Liang et al., 2022). Ni^2+^ Sepharose high performance was the product of GE healthcare. Menthol HPLC grade was purchased from Thermo Fisher Scientific. Other chemicals and solvents used were of analytical grade.

### Overexpression and Purification of WciN

The complete *wciN* gene of pneumococcus type 6B without terminator codon (GenBank: KT907353.1, 5253–6194) was synthesized and inserted into *Nde* I and *Xho* I of expression plasmid pET-28b by Sangon Biotech. The resulting recombinant plasmid pET-28b-*wciN* was then transformed into *E. coli* BL21 (DE3) competent cell for overexpression. The proper transformants were first grown at 37°C and 200 rpm in Luria-Bertani (LB) medium containing kanamycin (100 μg ml^−1^). When the cell density reached an OD value of 0.6–0.8 at 600 nm, isopropyl 1-thio-β-D-galactopyranoside (IPTG) (0.3 mM) was added to the cell culture for recombinant protein induction. After subsequent cultivation at 16°C for another 20 h, cells were harvested and disrupted by ultrasonic treatment. The resulting lysate was centrifuged and the supernatant was subjected to a nickel affinity chromatography for enzyme purification with three different buffers: equilibration buffer (50 mM Tris, 500 mM NaCl, 10 mM imidazole, and pH 7.5), washing buffer (50 mM Tris, 500 mM NaCl, 50 mM imidazole, and pH 7.5), and elution buffer (50 mM Tris, 500 mM NaCl, 200 mM imidazole, and pH 7.5). The purity and homogeneity of WciN protein were analyzed by 12.5% sodium dodecyl sulfate polyacrylamide gel electrophoresis (SDS-PAGE). Its concentration was measured by using a Thermo Scientific™ NanoDrop One spectrometer that was calibrated with the extinction coefficient predicted by ExPASy (http://web.expasy.org/protparam/). Finally, the purified enzyme was stored at -80°C containing 20% glycerol (*v/v*).

### Biochemical Characterization of WciN

The catalytic activity of the purified WciN was determined in a solution system as follows: 50 μg ml^−1^ of WciN, 5 mM MgCl_2_, 1 mM UDP-Gal, and 1 mM Glcα-PP-(CH_2_)_11_-OPh **1** in 50 mM buffer. Reaction mixtures were performed for 10 min and then terminated by boiling at 100°C for 30 s. After centrifuging for 10 min under 12,000 rpm, the supernatant was analyzed with HPLC (DionexCarboPac^TM^ PA-100 column, 4 × 250 mm, 0–1 M ammonium acetate buffer eluent). The byproduct UDP was monitored to assess the reaction process owing to its strong UV absorption at 260 nm and its convenience to be quantitated by HPLC (Wang et al., 2021; Liang et al., 2022).

The pH effect on enzyme activity was determined at pH values ranging from 6.0 to 10.5 with three different buffer systems at 37°C. The employed buffers included Bis-Tris-HCl (50 mM, pH 6.0, 6.5, and 7.0), Tris-HCl (50 mM, pH 7.0, 7.5, 8.0, 8.5, and 9.0), and Gly-NaOH (50 mM, pH 9.0, 9.5, 10.0, and 10.5). The optimal temperature for enzyme reaction was assessed at different temperatures (10, 16, 20, 25, 30, 37, 42, and 50°C) in 50 mM Gly-NaOH buffer (pH 9.0). To investigate the influence of metallic ions, enzyme activities were assayed in Gly-NaOH buffer (pH 9.0) with the presence of 5 mM following metal salts including ethylenediamine tetraacetic acid (EDTA), MgCl_2_, MnCl_2_, CaCl_2_, NiSO_4_, CoSO_4_, FeSO_4_, CuSO_4,_ and ZnSO_4_. To obtain the optimized Mg^2+^ concentration, the enzymatic reactions were carried out under varying concentrations of Mg^2+^ (0.3125–80 mM). Heat-treated WciN was served as a negative control. The Relative activity concluded from the pH and temperature test was defined as the relative value to the maximum enzyme activity, and the effect of metal ions on enzyme activity was determined using the activity measured without adding ions as the reference value.

For acceptor substrate specificity study, Glcα-PP-(CH_2_)_11_-OPh**,** Glcα-PP-(CH_2_)_7_-CH_3_, Glcα-P-(CH_2_)_11_-ONap, Glcβ-(CH_2_)_7_-CH_3,_ and Glcβ-(CH_2_)_11_-CH_3_ were examined with UDP-Gal as nucleotide donor, respectively. For donor substrate specificity study, UDP-Gal, UDP-Glc, UDP-GalNAc, UDP-GlcNAc, and UDP-GlcA were examined with Glcα-PP-(CH_2_)_11_-OPh as acceptor substrate, respectively. The reaction was performed in the optimized conditions for WciN.

### Site-Directed Mutagenesis of Key Amino Acids of WciN

Three single site mutated enzymes, namely WciN_D38N_, WciN_A150T_, and WciN_A150S_, were obtained using pET-28b-*wciN* plasmid as a template. Another two dual sites mutated enzymes, WciN_D38N/A150S_ and WciN_D38N/A150T_ were created using pET-28b-*wciN*_D38N_ as a template. All of the site mutations were carried out by the Fast Mutagenesis System (TransGen Biotech). Primers designed for corresponding amino acid substitution were listed in Supplementary Table S1. The mutant enzymes were overexpressed and purified following the similar protocols described earlier. Thereafter, UDP-Gal and UDP-Glc were applied to assay the donor recognition of the mutant enzymes, respectively. The reaction progress was monitored by thin-layer chromatography (TLC) or matrix-assisted laser desorption/ionization time of flight mass spectrometry (MALDI-TOF-MS). The developing solvent of TLC was a mixture of EtOAc/CH_3_OH/H_2_O/AcOH (*v/v/v/v*, 10/3/2/0.5), and the components on TLC were visualized by incubation at 180°C with a chromogenic solvent containing 93% ethyl alcohol, 3.5% sulfuric acid, 1% acetic acid, and 2.5% anisaldehyde.

To further investigate the effect of amino acid substitution at the position 150 of WciN on donor recognition, saturated mutation at A150 was systematically performed, and the corresponding oligonucleotide primers were shown in Supplementary Table S1. The catalytic activities of purified mutant enzymes were measured as described earlier. The molecular modeling of enzymes was conducted by PHYRE2 (http://www.sbg.bio.ic.ac.uk/phyre2/html/page.cgi?id = index).

### Enzyme Kinetics of WciN Mutants

The enzymatic reactions were carried out under the aforementioned optimized conditions, that is, in Gly-NaOH buffer (50 mM, pH 9.0) containing varying concentrations UDP-Gal/UDP-Glc and Glcα-PP-(CH_2_)_11_-OPh **1** with 5 mM MgCl_2_ at 37°C. Then, enzyme reactions using saturated UDP-sugar (4.0 mM) and varying concentrations of Glcα-PP-(CH_2_)_11_-OPh **1** (0.0625–4.0 mM) or Glcα-PP-(CH_2_)_11_-OPh **1** (1.0 mM) and varying concentrations of UDP-sugar (0.125–4.0 mM) were performed for 10 min. The Michaelis constant (*K*_m_) and maximal velocity (*V*_max_) values were graphed using the initial reaction velocities calculated from experimental data by GraphPad Prism 6.04 program.

### Milligram-Scale Enzymatic Synthesis of Disaccharides Galα1,3-Glcα-PP-(CH_2_)_11_-OPh 2 and Glcα1,3-Glcα-PP-(CH_2_)_11_-OPh 3

A 10 ml reaction system containing 2.4 mM UDP-Gal, 2 mM Glcα-PP-(CH_2_)_11_-OPh **1**, 5 mM MgCl_2_, and 100 μg ml^−1^ purified WciN_A150P_ in 50 mM Gly-NaOH buffer (pH 9.0) was incubated at 37°C for 1 h with gently shaking. After acceptor substrate **1** was completely converted into disaccharide product as monitored by TLC and MADI-TOF-MS analysis, the reaction was then terminated by boiling for 30 s. The reaction mixture was centrifuged at 12,000 rpm to remove the formed precipitate, and the resulting supernatant was freeze-dried, resuspended in methanol, and then filtered for further purification. The filtrate was purified by the reversed phase HPLC using a C18 column (10 × 250 mm) and gradient eluent (10–100% methanol in water containing 10 mM NH_4_HCO_3_). The fractions containing the desired product were pooled and concentrated to afford Galα1,3-Glcα-PP-(CH_2_)_11_-OPh **2** (12.7 mg, 85%) as a white solid. ^1^H NMR (600 MHz, CD_3_OD): δ 7.22 (t, 2H, *J* = 7.8 Hz, Ph), 6.89–6.84 (m, 3H, Ph), 5.65 (dd, 1H, *J* = 7.8, 3.6 Hz, H-1^Glc^), 5.34 (d, 1H, *J* = 2.4 Hz, H-1^Gal^), 4.24 (t, 1H, *J* = 6.0 Hz, H-5^Gal^), 4.00–3.56 (m, 7H, H-3,5^Glc^, H-4^Gal^, -OC*H*_2_CH_2_-, -CH_2_C*H*_2_OPh), 3.82 (dd, 1H, *J* = 12.0, 2.4 Hz, H-6a^Glc^), 3.80–3.75 (m, 2H, H-2,3^Gal^), 3.74 (dd, 1H, *J* = 11.4, 7.2 Hz, H-6a^Gal^), 3.66 (dd, 1H, *J* = 11.4, 3.6 Hz, H-6b^Gal^), 3.64 (dd, 1H, *J* = 12.0, 3.6 Hz, H-6b^Glc^), 3.52 (t, 1H, *J* = 9.6 Hz, H-4^Glc^), 3.47 (br d, 1H, *J* = 9.6 Hz, H-2^Glc^), 1.77–1.71 (m, 2H, -C*H*_2_CH_2_-), 1.67–1.61 (m, 2H, -C*H*_2_CH_2_-), 1.49–1.42 (m, 2H, -CH_2_C*H*_2_-), 1.41–1.27 (m, 15H, -CH_2_C*H*_2_-); ^13^C NMR (150 MHz, CD_3_OD): δ 159.16 (Ph), 128.93 (2C, Ph), 119.99 (Ph), 114.04 (2C, Ph), 99.52 (C-1^Gal^), 96.00 (d, *J*_C,P_ = 6.0 Hz, C-1^Glc^), 79.94 (C-3^Glc^), 73.19 (C-5^Glc^), 70.15 (d, *J*_C,P_ = 7.5 Hz, C-2^Glc^), 70.99 (C-5^Gal^), 70.46 (C-4^Glc^), 70.12 (C-3^Gal^), 69.79 (C-4^Gal^), 69.47 (C-2^Gal^), 67.14 (-CH_2_ *C*H_2_OPh), 65.94 (d, *J*_C,P_ = 6.0 Hz, -O*C*H_2_CH_2_-), 61.35 (C-6^Gal^), 61.11 (C-6^Glc^), 30.34 (d, *J* = 7.5 Hz, -OCH_2_ *C*H_2_CH_2_-), 29.34, 29.32, 29.28, 29.14, 29.12, 29.02, 25.76, 25.46 (8 C, -OCH_2_CH_2_(*C*H_2_)_8_CH_2_OPh); ^31^P NMR (243 MHz, CD_3_OD): δ -10.47 (d, *J* = 20.8 Hz) and -12.72 (d, *J* = 20.8 Hz); ESI-(-)-TOF HRMS *m/z*: calculated for C_29_H_49_O_18_P_2_ 747.2400 [M-H]^−^; found 747.2391.

A 10 ml reaction mixture of 50 mM Gly-NaOH buffer (pH 9.0) containing 2.4 mM UDP-Glc, 2 mM Glcα-PP-(CH_2_)_11_-OPh **1**, 5 mM MgCl_2_ and 200 μg ml^−1^ WciN_A150D_ was incubated at 37°C for 4 h. The reaction was then worked up following the same protocol as described earlier, yielding Glcα1,3-Glcα-PP-(CH_2_)_11_-OPh **3** (12.4 mg, 83%) as a white solid. ^1^H NMR (600 MHz, CD_3_OD): δ 7.22 (t, 2H, *J* = 7.8 Hz, Ph), 6.89–6.85 (m, 3H, Ph), 5.65 (dd, 1H, *J* = 7.8, 3.6 Hz, H-1^Glc^), 5.23 (d, 1H, *J* = 3.6 Hz, H-1^Glc′^), 4.01–3.90 (m, 6H, H-5^Glc^, H-5^Glc′^, -OC*H*_2_CH_2_-, -CH_2_C*H*_2_OPh), 3.88–3.80 (m, 3H, H-3^Glc,^ H-6a^Glc^, H-6a^Glc′^), 3.67 (t, 1H, *J* = 9.6 Hz, H-3^Glc′^), 3.65–3.58 (m, 2H, H-6b^Glc^, H-6b^Glc′^), 3.51 (t, 1H, *J* = 9.6 Hz, H-4^Glc^), 3.47 (br d, 1H, *J* = 9.6 Hz, H-2^Glc^), 3.40 (dd, 1H, *J* = 9.6, 3.6 Hz, H-2^Glc′^), 3.23 (t, 1H, *J* = 9.6 Hz, H-4^Glc′^), 1.77–1.71 (m, 2H, -C*H*_2_CH_2_-), 1.67–1.60 (m, 2H, -C*H*_2_CH_2_-), 1.49–1.42 (m, 2H, -CH_2_C*H*_2_-), 1.41–1.28 (m, 15H, -CH_2_C*H*_2_-); ^13^C NMR (150 MHz, CD_3_OD): δ 159.15 (Ph), 128.93 (2C, Ph), 119.99 (Ph), 114.04 (2C, Ph), 99.96 (C-1^Glc′^), 95.95 (d, *J*_C,P_ = 6.0 Hz, C-1^Glc^), 81.75 (C-3^Glc^), 73.78 (C-3^Glc′^), 73.06 (C-5^Glc^), 72.86 (C-2^Glc′^), 72.31 (C-5^Glc′^), 71.13 (d, *J*_C,P_ = 7.5 Hz, C-2^Glc^), 70.62 (C-4^Glc′^), 70.27 (C-4^Glc^), 67.41 (-*C*H_2_ *C*H_2_OPh), 65.86 (d, *J*_C,P_ = 6.0 Hz, -O*C*H_2_CH_2_-), 61.49 (C-6^Glc′^), 61.14 (C-6^Glc^), 30.36 (d, *J* = 7.5 Hz, -OCH_2_ *C*H_2_CH_2_-), 29.35, 29.32, 29.28, 29.15, 29.13, 29.02, 25.76, 25.48 (8 C, -OCH_2_CH_2_(*C*H_2_)_8_CH_2_OPh); ^31^P NMR (243 MHz, CD_3_OD): δ -10.40 (d, *J* = 20.8 Hz) and -12.68 (d, *J* = 20.8 Hz); ESI-(-)-TOF HRMS *m/z*: calculated for C_29_H_49_O_18_P_2_ 747.2400 [M-H]^−^; found 747.2394.

## Results and Discussion

### Overexpression and Purification of WciN

**FIGURE 2:**
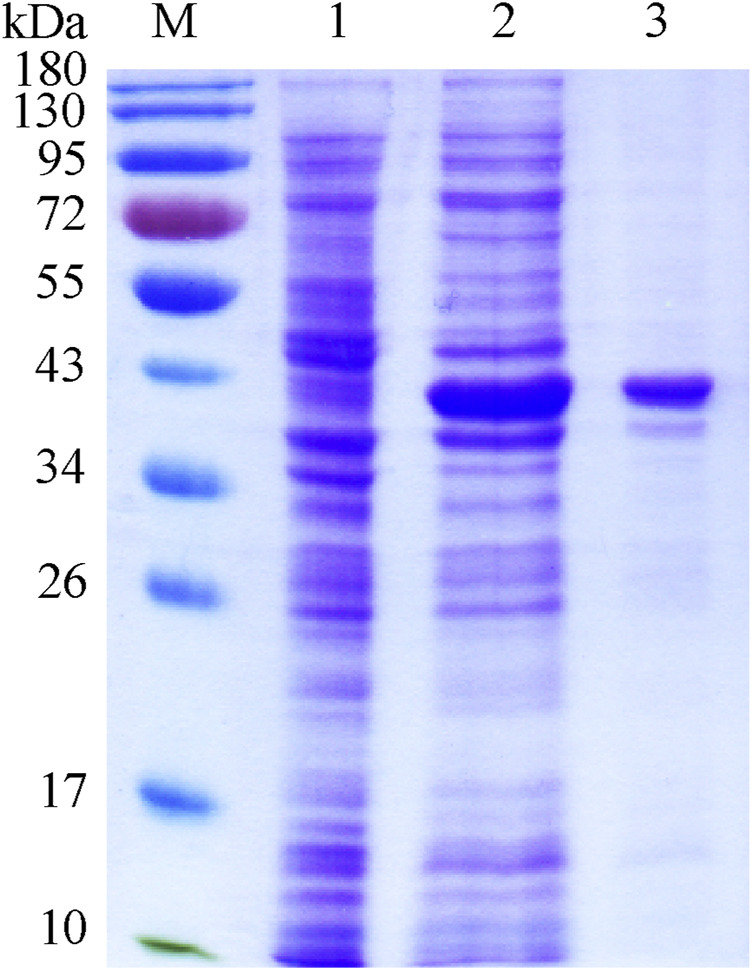
SDS-PAGE of recombinant WciN. *Lane M*, protein molecular weight standards; *lane 1*, whole *E. coli* BL21(DE3) cells with empty plasmid pET-28b; *lane 2*, crude extract of expression strain of WciN; *lane 3*, purified fusion protein His_6_-WciN-His_6_.

The recombinant plasmid pET-28b-*wciN* was designed to encode the full length of WciN with two His_6_ tags at its both N- and C-terminus for later convenient protein purification. The His_6_-WciN-His_6_ fusion protein was overexpressed and purified readily to homogeneity *via* Nickel-chelation affinity chromatography. The SDS-PAGE depicted in Figure 2 showed a distinct band at ∼40 kDa that was coincident with the theoretically calculated molecular weight (40.01 kDa) of recombinant WciN. In addition, its expression level was determined as 15.2 mg per liter using a NanoDrop One spectrophotometer.

### Biochemical Properties of the Recombinant WciN

**FIGURE 3:**
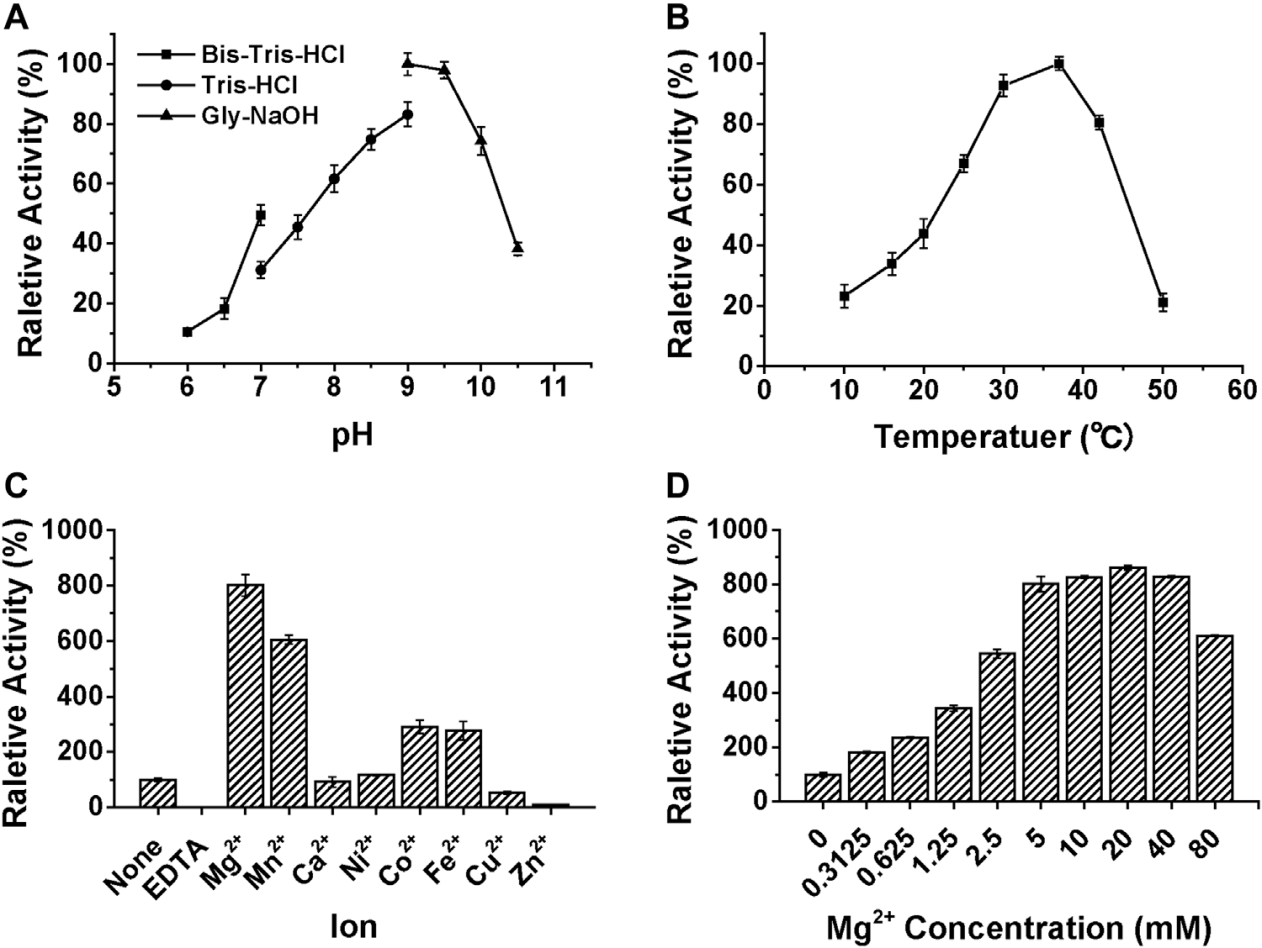
The influences of pH **(A)**, temperature **(B)**, ions **(C)**, and Mg^2+^ concentration **(D)** on the relative activity of WciN to catalyze galactosylation of Glcα-PP-(CH_2_)_11_-OPh **1** with UDP-Gal.

We have recently reported the optimized synthesis of the glycolipid Glcα-PP-(CH_2_)_11_-OPh **1** (Liang et al., 2022) and utilized it as an acceptor substrate to characterize several bacterial glycosyltransferases related to CPS RU biosynthesis (Wang et al., 2019; Wang et al., 2021; Liang et al., 2022). Therefore, using it as an enzymatic substrate, the detailed biochemical properties of WciN were then investigated. The enzymatic activities were analyzed by means of spectrophotometric analysis of the by-product UDP, and the results were shown in Figure 3. The better activity (>60%) of WciN was observed under weak alkali conditions (pH 8.0–10.0), and the optimal pH value for its activity was 9.0 (Figure 3A). WciN enzyme was highly active (>80%) from 30 to 42°C, and the best glycosylation activity was determined at 37°C (Figure 3B). Furthermore, the existence of EDTA could completely inhibit enzyme activity (Figure 3C), indicating WciN might belong to GT-A glycosyltransferase family (Lairson et al., 2008; Han et al., 2012). Among eight tested divalent cation ions, Cu^2+^ and Zn^2+^ obviously reduced enzyme activity, and Ca^2+^ and Ni^2+^ slightly affected its activity, whilst Mg^2+^, Mn^2+^, Co^2+^, and Fe^2+^ exhibited remarkable promoting ability on enzyme activity (Figure 3C). It has been disclosed that a 5∼8 fold improvement of WciN activity was achieved in the presence of Mn^2+^ or Mg^2+^. In addition, the influence of Mg^2+^ concentrations on WciN activity was also examined. As shown in Figure 3D, the activity of WciN improved sharply under a broad range of 5–40 mM of Mg^2+^ concentrations but without any significant difference. Collectively, the optimal reaction conditions for WciN enzyme were established to be 5 mM Mg^2+^ in 50 mM Gly-NaOH buffer with pH 9.0 at 37°C.

### Acceptor Substrate Specificity of WciN

The specificity of WciN toward five acceptor substrates was investigated with the earlier optimized reaction conditions using UDP-Gal as the nucleotide donor (Table 1). The enzymatic reactions were monitored by TLC and HRMS (Supplementary Figure S1). Among five sugar acceptors, only Glcα-PP-(CH_2_)_11_-OPh and Glcα-PP-(CH_2_)_7_-CH_3_, which had diphosphate moiety in structure, could be well recognized by WciN, whereas Glcα-P-(CH_2_)_11_-ONap with monophosphate moiety and the other two acceptors, Glcβ-(CH_2_)_11_-CH_3_ and Glcβ-(CH_2_)_7_-CH_3_, without any phosphate moiety exhibited none detectable activity. These results indicated that the diphosphate moiety in the acceptor substrate played an important role in the acceptor recognition of WciN.

**TABLE 1:** Investigation of acceptor recognition of WciN.

No.	Acceptor	Structure	Activity
1	Glcα-PP-(CH_2_)_11_-OPh	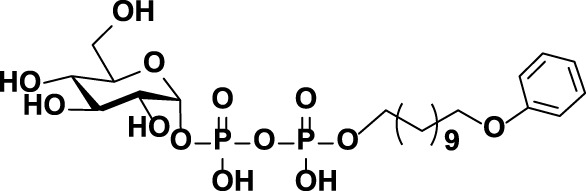	+
2	Glcα-PP-(CH_2_)_7_-CH_3_	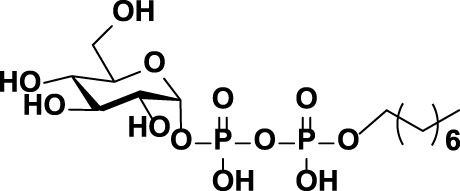	+
3	Glcα-P-(CH_2_)_11_-ONap	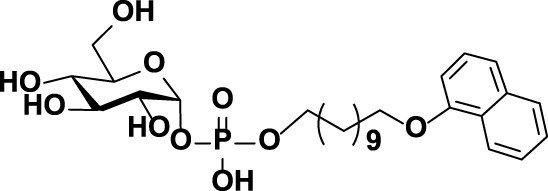	−
4	Glcβ-(CH_2_)_7_-CH_3_	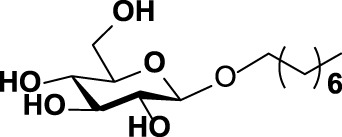	−
5	Glcβ-(CH_2_)_11_-CH_3_	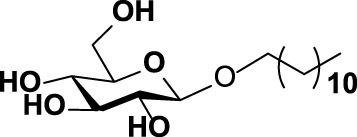	−

+, detectable activity by TLC and HRMS; −, no detectable activity.

### Nucleotide Donor Recognition of WciN Enzyme and Its Mutants

**FIGURE 4:**
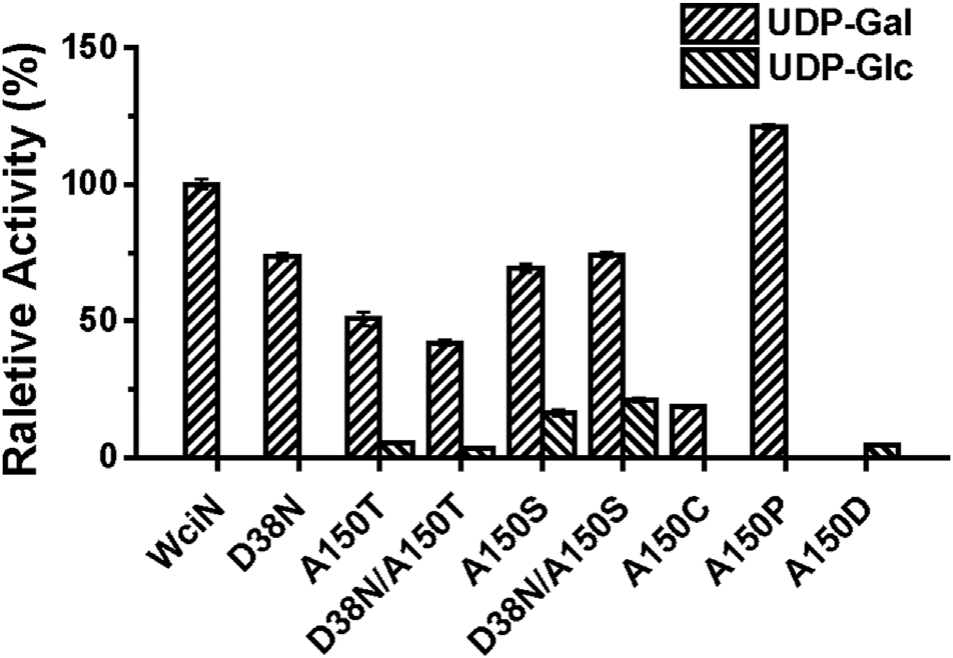
Relative activities of WciN mutant enzymes toward UDP-Gal and UDP-Glc donors.

In a previous study, Nahm and co-workers have disclosed that the amino acid replacement at the position 150 and/or 38 of WciN could alter donor substrate specificities, resulting in the emergence of two new hybrid serotypes 6F and 6G (Oliver et al., 2013). To further confirm this conclusion, five mutant enzymes including WciN_D38N_, WciN_A150T_, WciN_A150S_, WciN_D38N/A150T_, and WciN_D38N/A150S_ were accordingly designed, overexpressed, and purified to homogeneity. Using UDP-Gal and UDP-Glc as sugar nucleotide donors, the activities of these mutant enzymes were detected with TLC (Supplementary Figures S2A,B) and then analyzed by HPLC (Figure 4). Compared to wild-type WciN, all mutated enzymes could well recognize UDP-Gal donor (Supplementary Figure S2A) but exhibit reduced galactosylation activities in different degrees (40–80% relative activities) (Figure 4). Interestingly, except WciN_D38N_ mutant, all other four WciN mutants could also accept UDP-Glc donor and showed weak to the good catalytic ability for glucosylation (Supplementary Figure S2B). Moreover, the glucosylation abilities of WciN_A150S_ and WciN_D38N/A150S_ mutants were significantly higher than those exerted by WciN_A150T_ and WciN_D38N/A150T_ mutants (Figure 4). These aforementioned results disclosed that the A150 residue of WciN was the pivotal residue responsible for nucleotide donor recognition and its mutation could alter nucleotide donor recognition, whereas mutation of the D38 residue could only decrease the enzymatic activity but not affect its donor specificity. All these findings almost coincided with the results reported previously (Oliver et al., 2013).

Thereafter, saturated mutation on A150 residue of WciN was executed using the designed primers listed in Supplementary Table S1. Except WciN_A150T_ and WciN_A150S_, other seventeen mutants were obtained and their enzymatic activities were accordingly examined using the same protocol described earlier. As shown in Supplementary Figures S2C,D, WciN_A150C_ and WciN_A150P_ mutants exhibited the catalytic activity to only recognize UDP-Gal as donor substrate with increased activity for WciN_A150P_ (121% relative activity) and a decreased activity for WciN_A150C_ (19% relative activity), whereas WciN_A150D_ showed the capability to only accept UDP-Glc as donor substrate even with a lower glucosylation activity (∼5% relative activity, Figure 4). Nevertheless, the rest mutated enzymes did not exert any detectable enzymatic activities toward UDP-Gal or UDP-Glc. All these results suggested that rational residue replacement at position 150 of WciN could affect the recognizable capability against donor substrate or change its donor specificity. In addition, WciN and its active mutants could not recognize other UDP-sugars, such as UDP-GlcNAc, UDP-GalNAc, and UDP-GlcA, indicating their relative donor specificity. Incidentally, the acceptor specificity of these mutants coincided well with that of wild type WciN enzyme.

In order to explore how the residue replacement at Ala150 of WciN affected its enzyme activity, molecular modeling of WciN and its mutants WciN_A150T_ and WciN_A150P_ were conducted using LgtC, a retaining galactosyltransferase from *Neisseria meningitides*, as the modeling template (Persson et al., 2001). The Gln189 residue of LgtC was located at the catalytic center and interacted with nucleotide donor through *van der* Waals, whilst interacted with acceptor substrate with the assistant of Ala154 residue through several hydrogen bonds (Persson et al., 2001). Alignment of the amino acid sequence of WciN with that of LgtC indicated that Ala150 and Leu185 residues of WciN corresponded to Ala154 and Gln189 residues of LgtC, respectively (Supplementary Figure S3). Therefore, it indicated that the residues Ala150 of WciN indirectly influenced substrate recognition by affecting its Leu185 residue.

### Enzyme Kinetics of WciN Enzyme and Its Mutants

**FIGURE 5:**
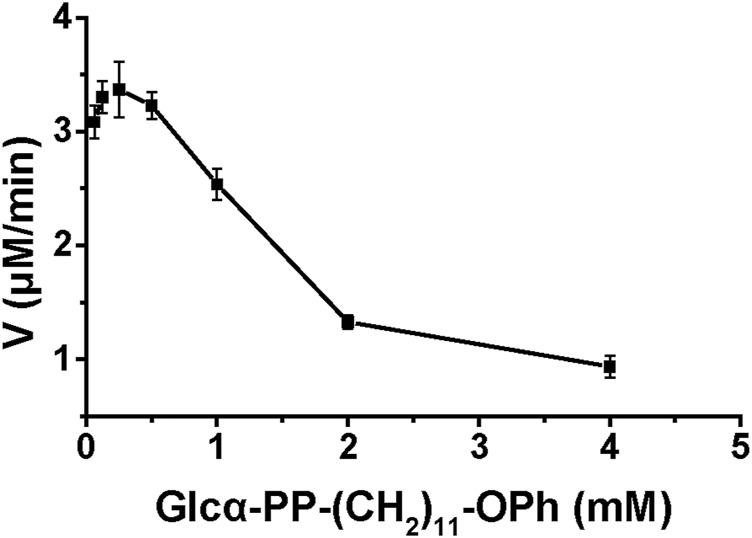
Influence of acceptor concentration on enzyme reaction velocity.

The enzyme kinetics of WciN and its mutants were examined at optimal reaction conditions established earlier. The influence of acceptor substrate concentration on the enzyme activity of WciN was explored first. As depicted in Figure 5, the reaction velocity catalyzed by WciN was dramatically reduced as the concentration of Glcα-PP-(CH_2_)_11_-OPh **1** was greater than 0.25 mM, indicating the activity of WciN could be easily inhibited even by a slightly higher concentration of acceptor substrate. This finding was radically different from those reported glycosyltransferases that could well accept Glcα-PP-(CH_2_)_11_-OPh **1** as substrate acceptor (Wang et al., 2019; Liang et al., 2022). Bioinformatics analysis of the amino acid sequence of WciN revealed that there was no transmembrane domain in WciN, lacking a domain that interacts with the membrane. Thus, the great difference in recognizing acceptor substrates by WciN might be remarkably affected by the variation of substrate micelles formed in aqueous solution due to the amphipathic character of Glcα-PP-(CH_2_)_11_-OPh **1**. Therefore, the enzyme kinetics of WciN and its mutants, WciN_A150P_ and WciN_A150D_, toward nucleotide donor (UDP-Gal or UDP-Glc) were then briefly measured using 0.125–4.0 mM UDP-Gal/UDP-Glc and Glcα-PP-(CH_2_)_11_-OPh **1** at suitable concentration (1.0 mM). The *K*_m_ and *V*_max_ values were calculated from Michaelis–Menten plots and listed in Table 2.

**TABLE 2:** Enzyme kinetics using UDP-Gal and UDP-Glc as donor.

Enzyme	UDP-Gal		UDP-Glc	
	*K*_m_ (mM)	*V*_max_ (μM/min)	*K*_m_ (mM)	*V*_max_ (μM/min)
WciN	5.5 ± 0.33	133.5 ± 5.28	—	—
WciN_A150P_	7.06 ± 0.78	151.8 ± 11.79	—	—
WciN_A150D_	—	—	3.52 ± 1.18	0.39 ± 0.08

—, no data.

### Preparation of Disaccharide Products 2 and 3

**SCHEME 1:**
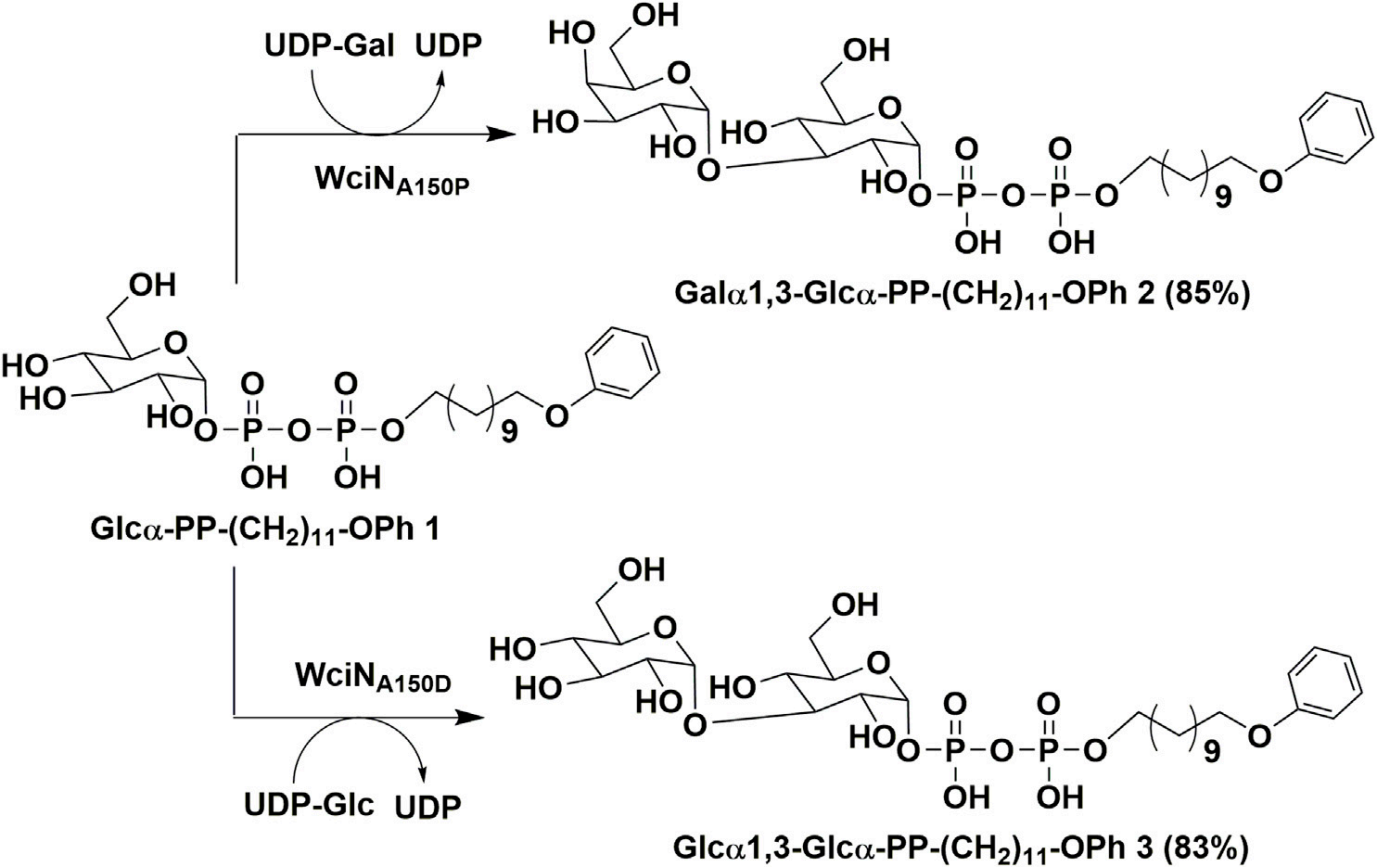
Enzymatic synthesis of Galα1,3-Glcα-PP-(CH_2_)_11_-OPh **2** and Glcα1,3-Glcα-PP-(CH_2_)_11_-OPh **3**.

As outlined in Scheme 1, using Glcα-PP-(CH_2_)_11_-OPh **1** as substrate acceptor, the disaccharide products Galα1,3-Glcα-PP-(CH_2_)_11_-OPh **2** and Glcα1,3-Glcα-PP-(CH_2_)_11_-OPh **3** were efficiently prepared under the earlier optimized reaction conditions by WciN_A150P_ and WciN_A150D_ mutants, respectively. Each enzymatic reaction proceeded smoothly and was monitored timely by TLC analysis, and terminated within 1–4 h after the full consumption of **1**. The disaccharides **2** and **3** were then obtained in milligram quantities and high yields of 83–85% after semi-preparative HPLC purification. Furthermore, the correct stereo-/regio-selectivity of each disaccharide product functioned by the WciN mutant was well verified with the assistance of the 1D and 2D NMR spectra. The small ^3^ *J*_1,2_ coupling constants (2.4 Hz for **2**; 3.6 Hz for **3**) of doublet peaks of the H-1^Gal/Glc′^ signals in their ^1^H NMR spectra indicated the new formation of α-glyosidic bonds, whilst the observed correlation signals of C-1^Gal^/H-3^Glc^ and H-1^Glc′^/C-3^Glc^ in their gHMBC spectra confirmed the regioselective formation of the 1,3-glycosidic linkages.

## Conclusion

In this study, the detailed biological characterization of WciN from pneumococcus type 6B strain was investigated *in vitro*. The single or dual site-directed mutagenesis with D38 and A150 residues of WciN followed by comparison of glycosylation activities of the resultant mutant enzymes revealed that A150 residue played the pivotal role in altering donor recognition (Oliver et al., 2013). Accordingly, saturated mutation at position 150 of WciN was implemented, and only WciN_A150T_, WciN_A150S_, WciN_A150C_, WciN_A150P_, and WciN_A150D_ mutants exhibited the catalytic activity in different degree. Among them, WciN_A150T_ and WciN_A150S_ mutants were recognized as bi-specific glycosyltransferases that could catalyze both galactosylation and glucosylation. Furthermore, WciN_A150P_ mutant showed remarkably increased capability (121% relative activity) for recognition on UDP-Gal with comparison to that of wild WciN enzyme, whereas WciN_A150D_ mutant completely abolished the recognition capability toward UDP-Gal, but could accept UDP-Glc as sole donor substrate even with very low activity (∼5% relative activity). All these findings indicated that the single site-mutation of galactosyltransferase WciN at A150 residue could cause the different recognition toward nucleotide donor and thus trigger complete pneumococcus serotype switch. Finally, using WciN_A150P_ and WciN_A150D_ mutants as useful tool enzymes, disaccharide products Galα1,3Glcα-PP-(CH_2_)_11_-OPh **2** and Glcα1,3Glcα-PP-(CH_2_)_11_-OPh **3** were successfully achieved in multi-milligram scale.

## Data Availability

The datasets presented in this study can be found in online repositories. The names of the repository/repositories and accession number(s) can be found in the article/Supplementary Material.
