# Supplementary material for: Biochemical Characterization and Synthetic Application of WciN and Its Mutants From Streptococcus pneumoniae Serotype 6B
Source: Front Chem. 2022 Jun 15;10:914698. doi: 10.3389/fchem.2022.914698 (PMC9240355; doi:10.3389/fchem.2022.914698)
Supplement: Supplementary file 1 [file DataSheet1.pdf]

Supplementary Material

Table S1. Oligonucleotide primers used to amino acid substitution in this study

Name	Primer sequence (5’ → 3’)
WciN$_{D38N}$-F	TTATGGATTATTGCT*AA*TAAAGTTTC
WciN$_{D38N}$-R	*T*AGCAATAATCCATAAATTTAAATC
WciN$_{A150T}$-F	AAACCAATGTTTAAT*AC*TGGAGTTAT
WciN$_{A150T}$-R	*T*ATTAAACATTGGTTTGTCGATTGG
WciN$_{A150S}$-F	AAACCAATGTTTAAT*TC*TGGAGTTAT
WciN$_{A150S}$-R	*A*ATTAAACATTGGTTTGTCGATTGG
WciN$_{A150G}$-F	AAACCAATGTTTAATG*G*TGGAGTTAT
WciN$_{A150G}$-R	*C*CATTAAACATTGGTTTGTCGATTGG
WciN$_{A150V}$-F	AAACCAATGTTTAAT*GT*TGGAGTTAT
WciN$_{A150V}$-R	*AC*ATTAAACATTGGTTTGTCGATTGG
WciN$_{A150L}$-F	AAACCAATGTTTAAT*CT*TGGAGTTAT
WciN$_{A150L}$-R	*AG*ATTAAACATTGGTTTGTCGATTGG
WciN$_{A150I}$-F	AAACCAATGTTTAAT*AT*TGGAGTTAT
WciN$_{A150I}$-R	*AT*ATTAAACATTGGTTTGTCGATTGG
WciN$_{A150C}$-F	AAACCAATGTTTAAT*TG*TGGAGTTAT
WciN$_{A150C}$-R	*CA*ATTAAACATTGGTTTGTCGATTGG
WciN$_{A150M}$-F	AAACCAATGTTTAAT*ATG*GGAGTTAT
WciN$_{A150M}$-R	*CAT*ATTAAACATTGGTTTGTCGATTGG
WciN$_{A150D}$-F	AAACCAATGTTTAATG*A*TGGAGTTAT
WciN$_{A150D}$-R	*T*CATTAAACATTGGTTTGTCGATTGG
WciN$_{A150E}$-F	AAACCAATGTTTAATG*AA*GGAGTTAT
WciN$_{A150E}$-R	*TT*CATTAAACATTGGTTTGTCGATTGG
WciN$_{A150N}$-F	AAACCAATGTTTAAT*AA*TGGAGTTAT
WciN$_{A150N}$-R	*TT*ATTAAACATTGGTTTGTCGATTGG
WciN$_{A150Q}$-F	AAACCAATGTTTAAT*CAA*GGAGTTAT
WciN$_{A150Q}$-R	*TTG*ATTAAACATTGGTTTGTCGATTGG
WciN$_{A150K}$-F	AAACCAATGTTTAAT*AAA*GGAGTTAT
WciN$_{A150K}$-R	*TTT*ATTAAACATTGGTTTGTCGATTGG
WciN$_{A150R}$-F	AAACCAATGTTTAAT*CG*TGGAGTTAT

WciN$_{A150R}$-R	*CG*ATTAAACATTGGTTTGTCGATTGG
WciN$_{A150H}$-F	AAACCAATGTTTAAT*CA*TGGAGTTAT
WciN$_{A150H}$-R	*TG*ATTAAACATTGGTTTGTCGATTGG
WciN$_{A150P}$-F	AAACCAATGTTTAAT*CC*TGGAGTTAT
WciN$_{A150P}$-R	*G*ATTAAACATTGGTTTGTCGATTGG
WciN$_{A150F}$-F	AAACCAATGTTTAAT*TT*TGGAGTTAT
WciN$_{A150F}$-R	*AA*ATTAAACATTGGTTTGTCGATTGG
WciN$_{A150Y}$-F	AAACCAATGTTTAAT*TA*TGGAGTTAT
WciN$_{A150Y}$-R	*TA*ATTAAACATTGGTTTGTCGATTGG
WciN$_{A150W}$-F	AAACCAATGTTTAAT*TGG*GGAGTTAT
WciN$_{A150W}$-R	*CCA*ATTAAACATTGGTTTGTCGATTGG

The underline marked the codons of substituted amino acid, the italics showed the changed bases.

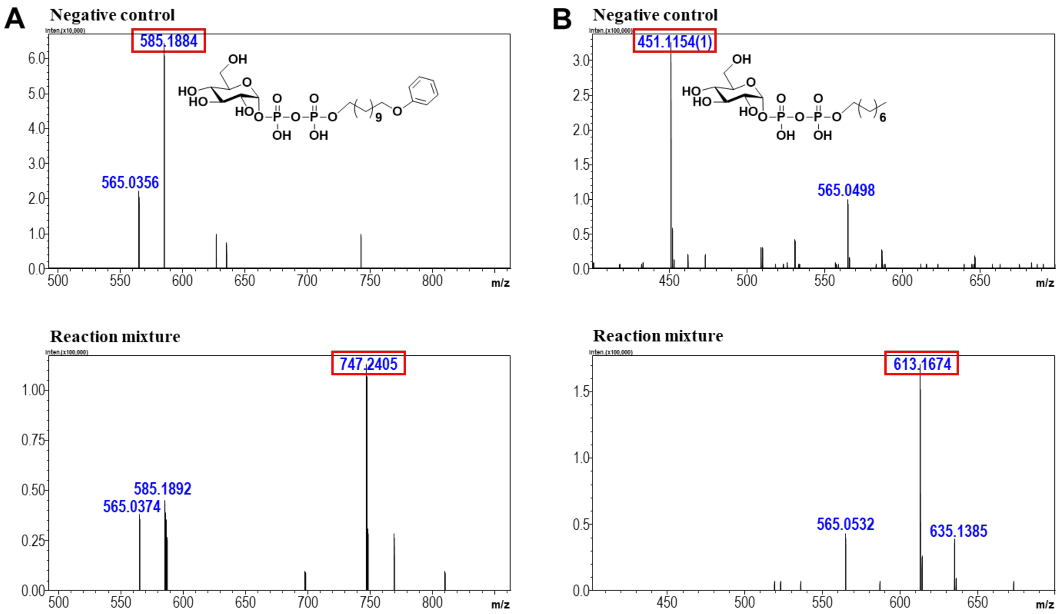

Figure S1. The HRMS results of acceptor substrate specificity of WciN with Glcα-PP-$(CH_2)_{11}$-OPh (A) and Glcα-PP-$(CH_2)_7$-CH_3 (B).

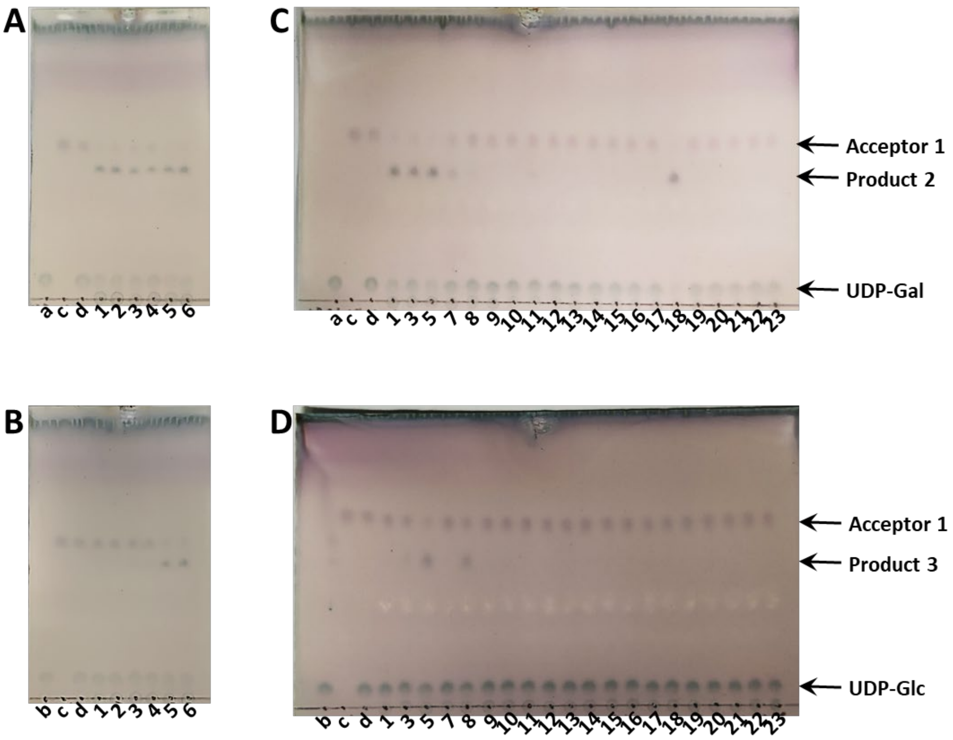

Figure S2. TLC results of recognition of UDP-Gal (A and C) and UDP-Glc (B and D) by mutant enzymes. a, UDP-Gal; b, UDP-Glc; c, Glcα-PP-$(CH_2)_{11}$-OPh; d, control reaction with heat-treated WciN; 1-23, reaction with WciN and mutants (1, WciN; 2, WciN$_{D38N}$; 3, WciN$_{A150T}$; 4, WciN$_{D38N/A150T}$; 5, WciN$_{A150S}$; 6, WciN$_{D38N/A150S}$; 7, WciN$_{A150C}$; 8, WciN$_{A150D}$; 9, WciN$_{A150E}$; 10, WciN$_{A150F}$; 11, WciN$_{A150G}$; 12, WciN$_{A150H}$; 13, WciN$_{A150I}$; 14, WciN$_{A150K}$; 15, WciN$_{A150L}$; 16, WciN$_{A150M}$; 17, WciN$_{A150N}$; 18, WciN$_{A150P}$; 19, WciN$_{A150Q}$; 20, WciN$_{A150R}$; 21, WciN$_{A150V}$; 22, WciN$_{A150W}$; 23, WciN$_{A150Y}$).

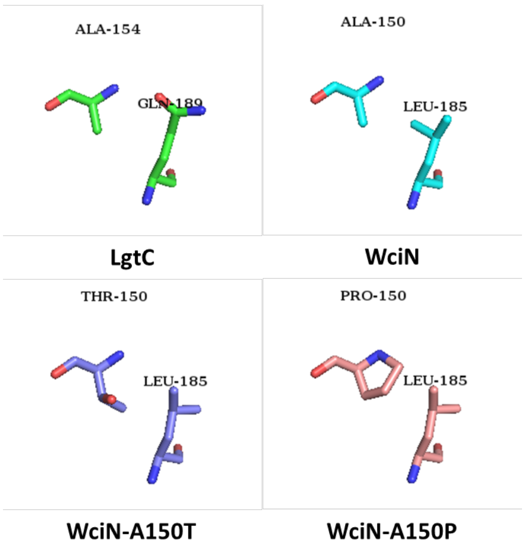

Figure S3. Partial enlarged detail of modeling results of LgtC, WciN and mutant enzymes.

ESI-HRMS Spectra of disaccharide 2 and 3

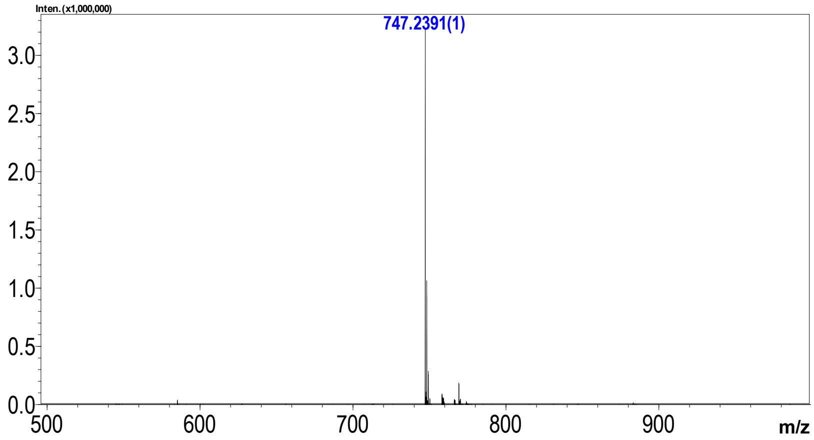

ESI-(-)-TOF HRMS of Galα1,3-Glcα-PP-$(CH_2)_{11}$-OPh **2**

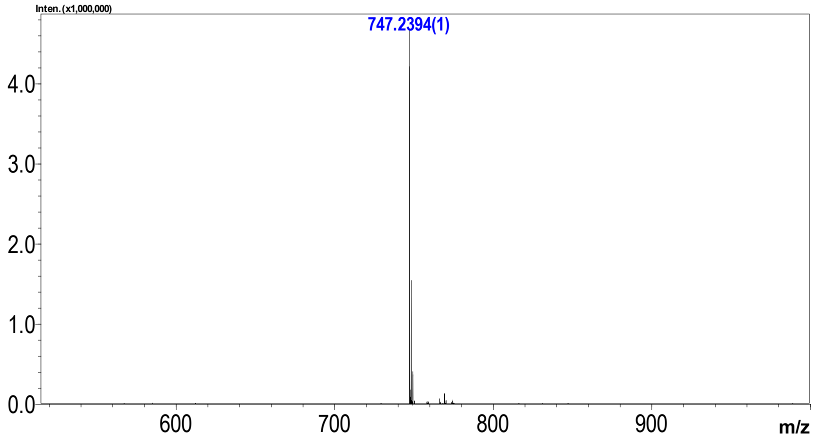

ESI-(-)-TOF HRMS of Glcα1,3-Glcα-PP-$(CH_2)_{11}$-OPh **3**

NMR spectra of Galα1,3-Glcα-PP-$(CH_2)_{11}$-OPh 2

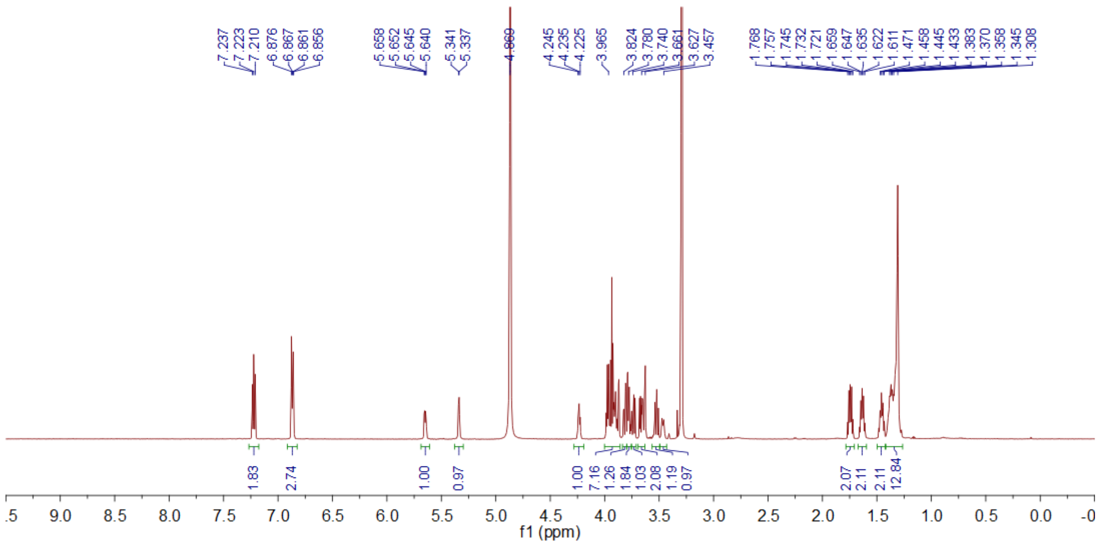

^{1}H NMR spectrum of Galα1,3-Glcα-PP-$(CH_2)_{11}$-OPh **2** (CD_3OD, 600 MHz, 25 °C)

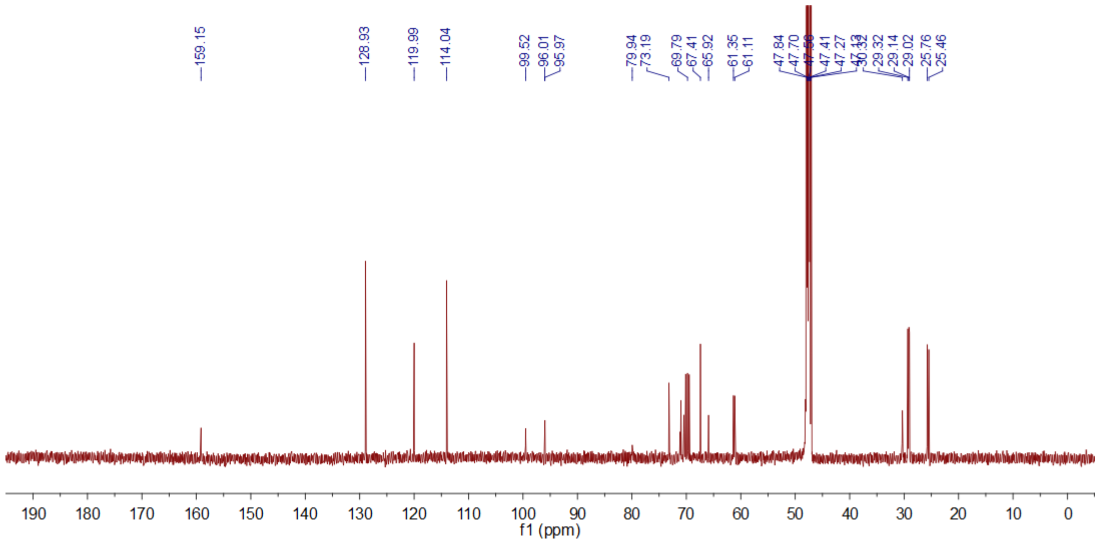

^{13}C NMR spectrum of Galα1,3-Glcα-PP-$(CH_2)_{11}$-OPh **2** (CD_3OD, 150 MHz, 25 °C)

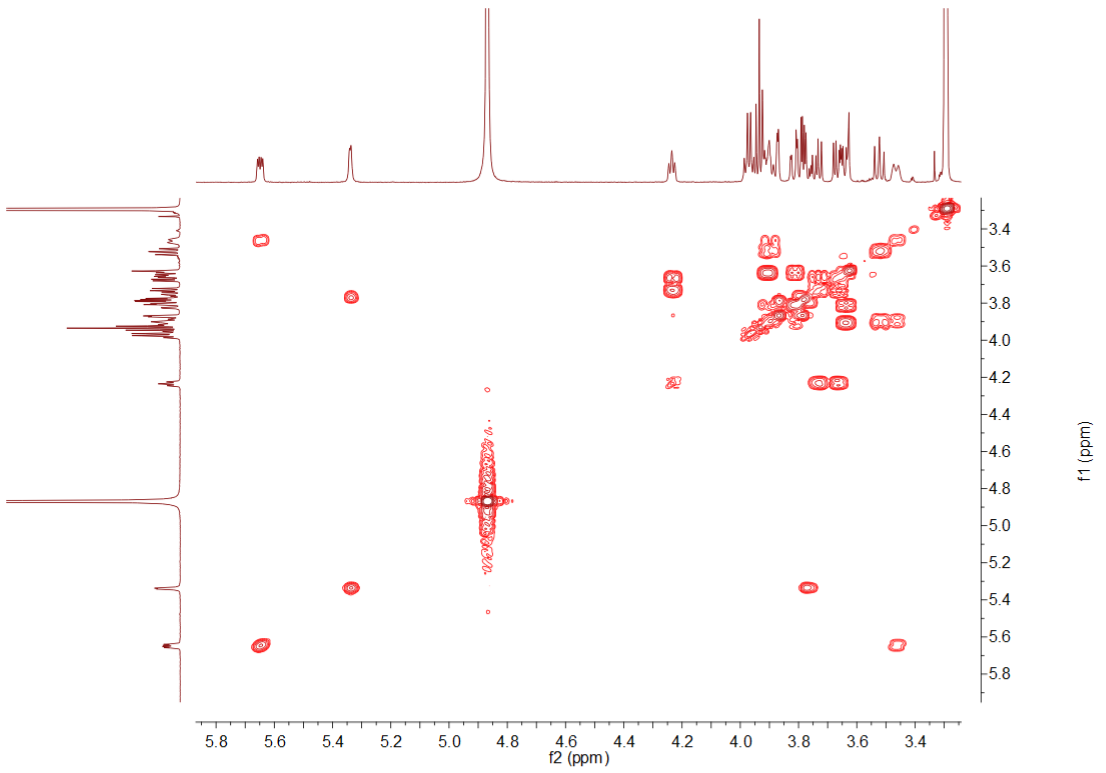

gCOSY spectrum of Galα1,3-Glcα-PP-$(CH_2)_{11}$-OPh **2** (CD_3OD, 600/600 MHz, 25 ºC)

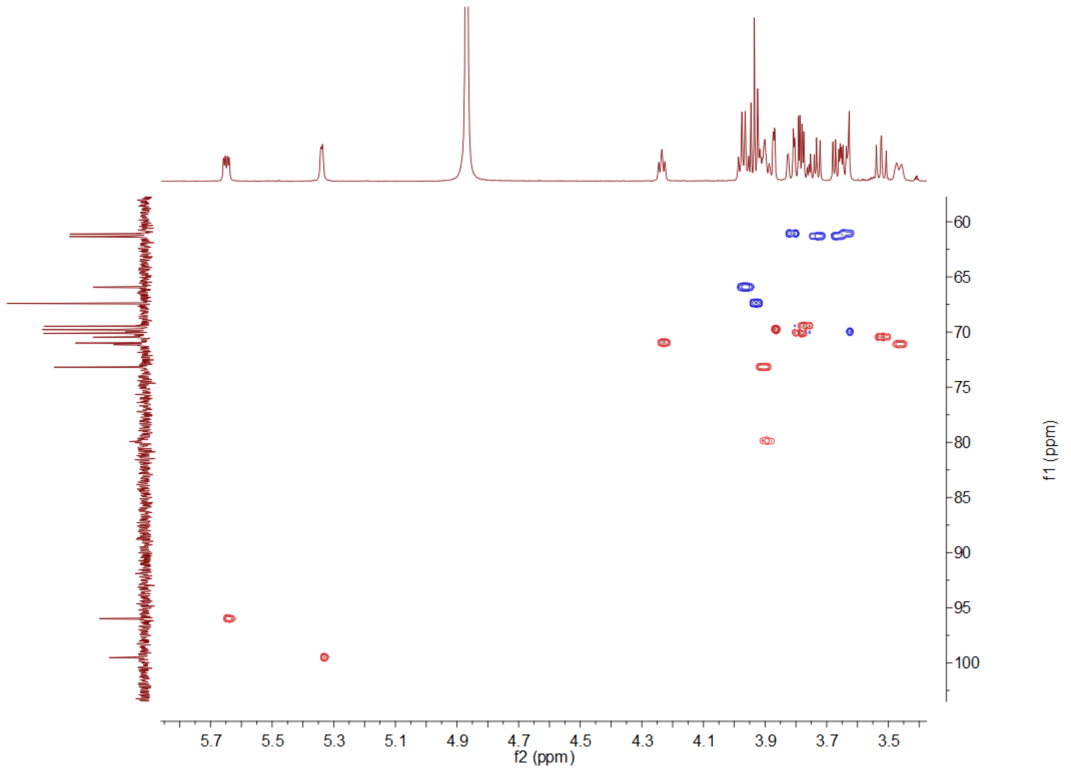

gHSQC spectrum of Galα1,3-Glcα-PP-$(CH_2)_{11}$-OPh **2** (CD_3OD, 600/150 MHz, 25 ºC)

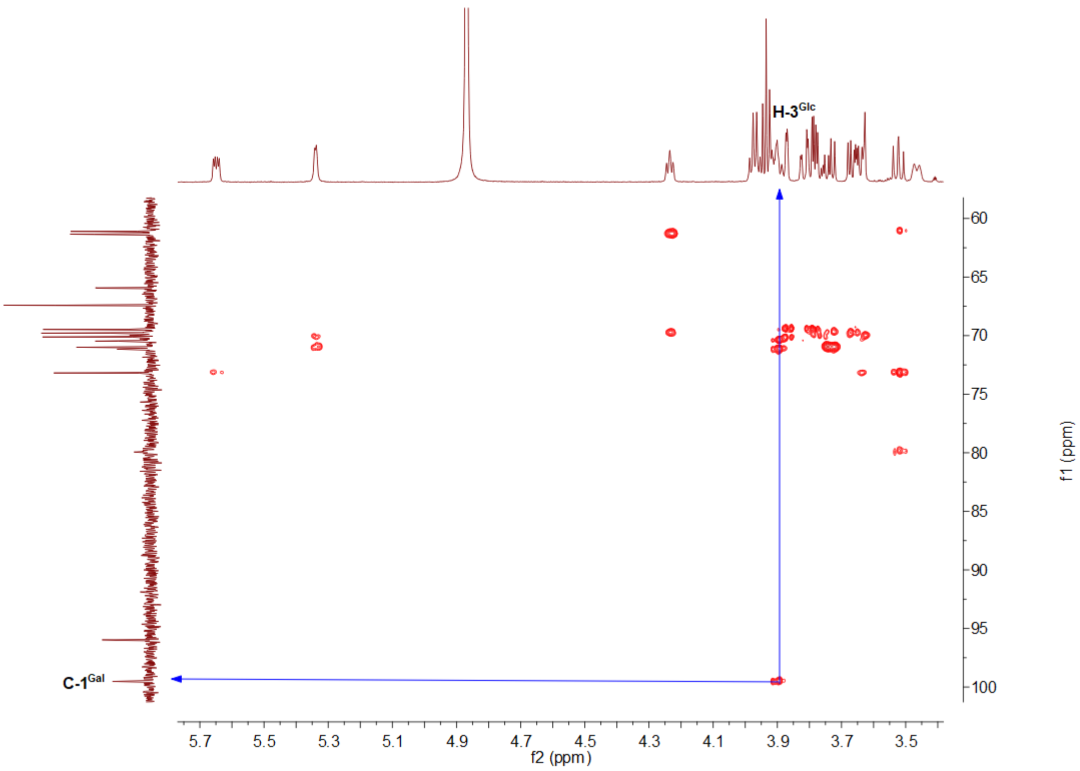

gHMBC spectrum of Galα1,3-Glcα-PP-(CH$_2$)$_{11}$-OPh **2** (CD$_3$OD, 600/150 MHz, 25 °C)

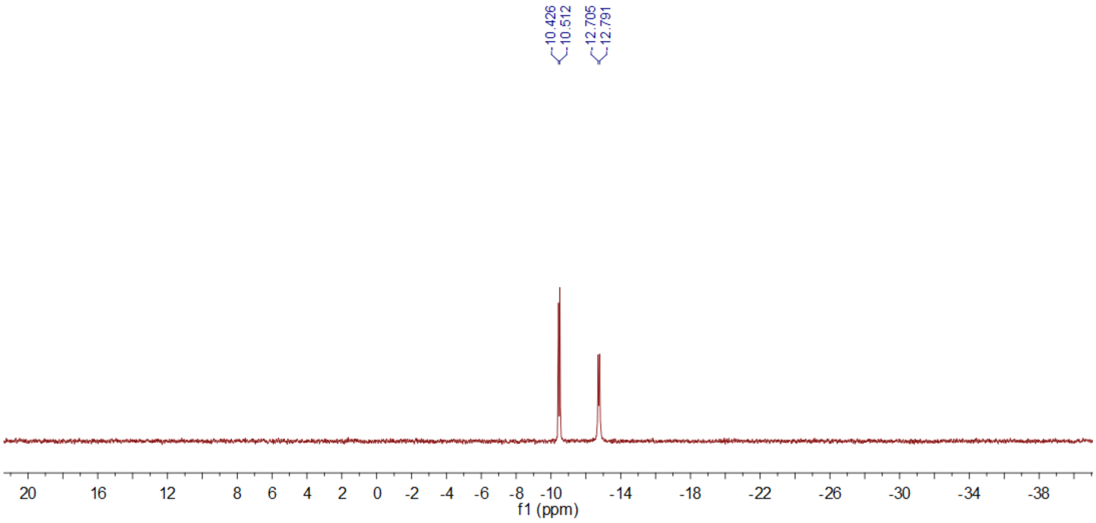

^{31}P NMR spectrum of Galα1,3-Glcα-PP-(CH$_2$)$_{11}$-OPh **2** (CD$_3$OD, 243 MHz, 25 °C)

NMR spectra of Glcα1,3-Glcα-PP-$(CH_2)_{11}$-OPh 3

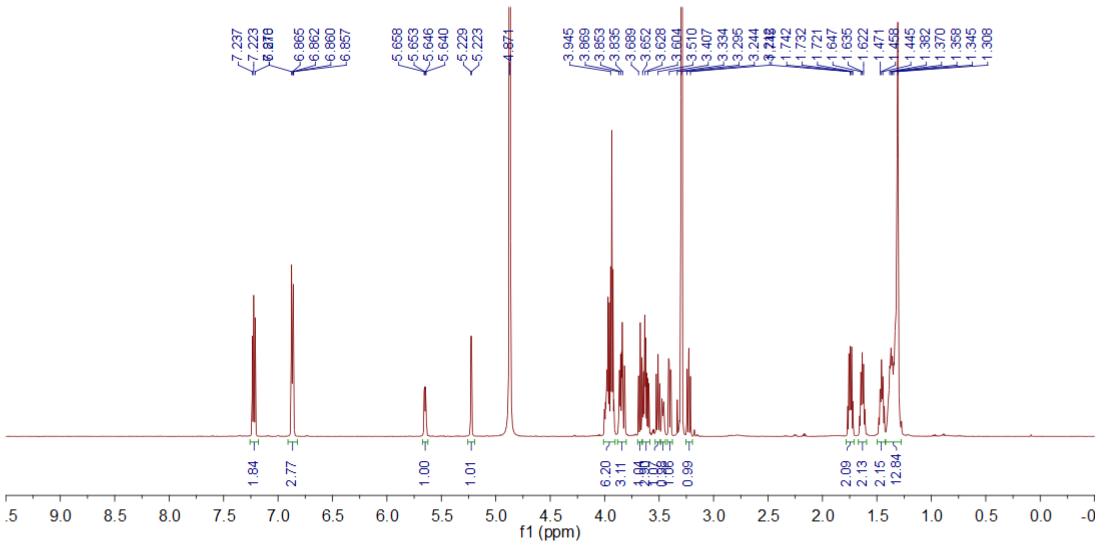

^{1}H NMR spectrum of Glcα1,3-Glcα-PP-$(CH_2)_{11}$-OPh **3** (CD_3OD, 600 MHz, 25 ºC)

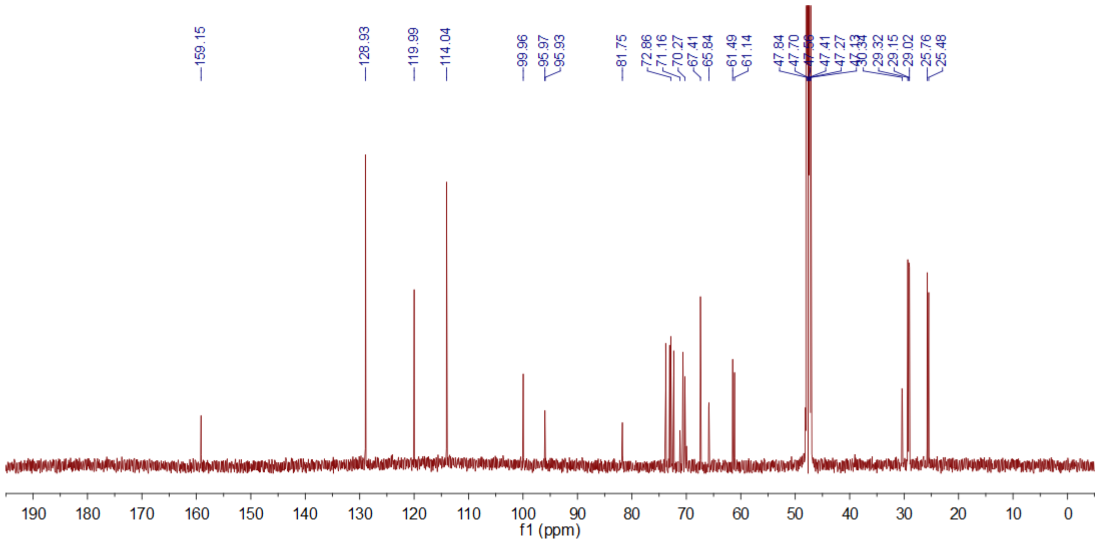

^{13}C NMR spectrum of Glcα1,3-Glcα-PP-$(CH_2)_{11}$-OPh **3** (CD_3OD, 150 MHz, 25 ºC)

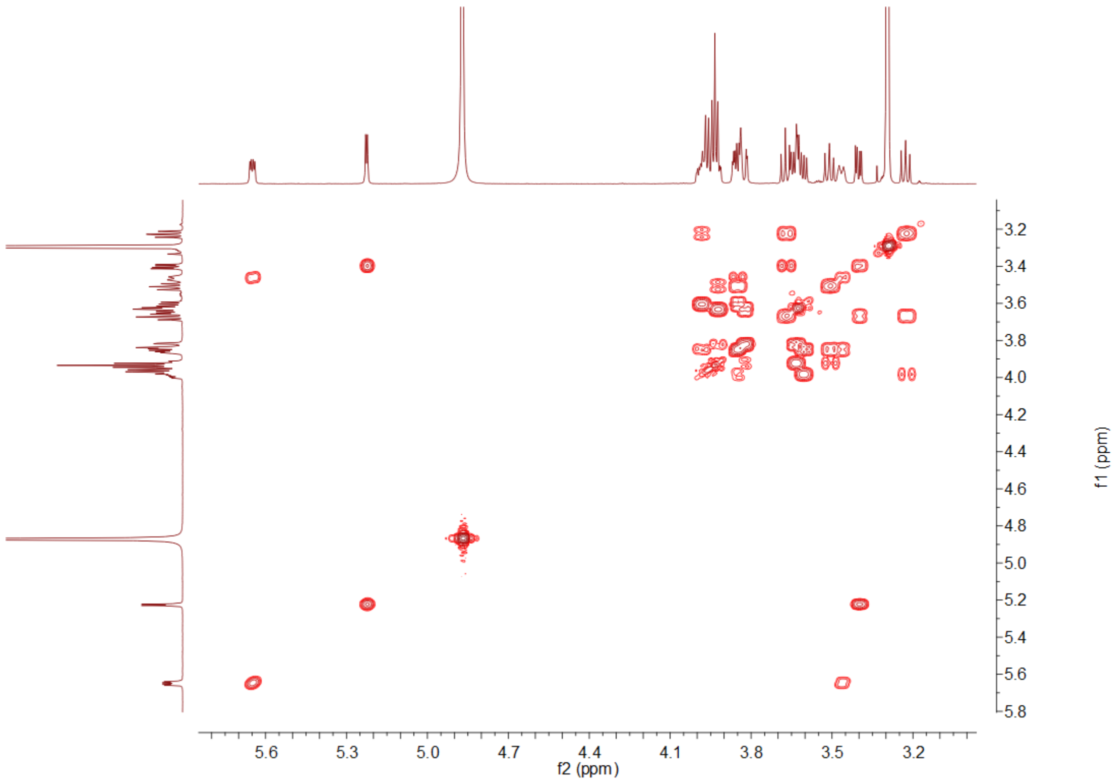

gCOSY spectrum of Glcα1,3-Glcα-PP-$(CH_2)_{11}$-OPh **3** (CD_3OD, 600/600 MHz, 25 ºC)

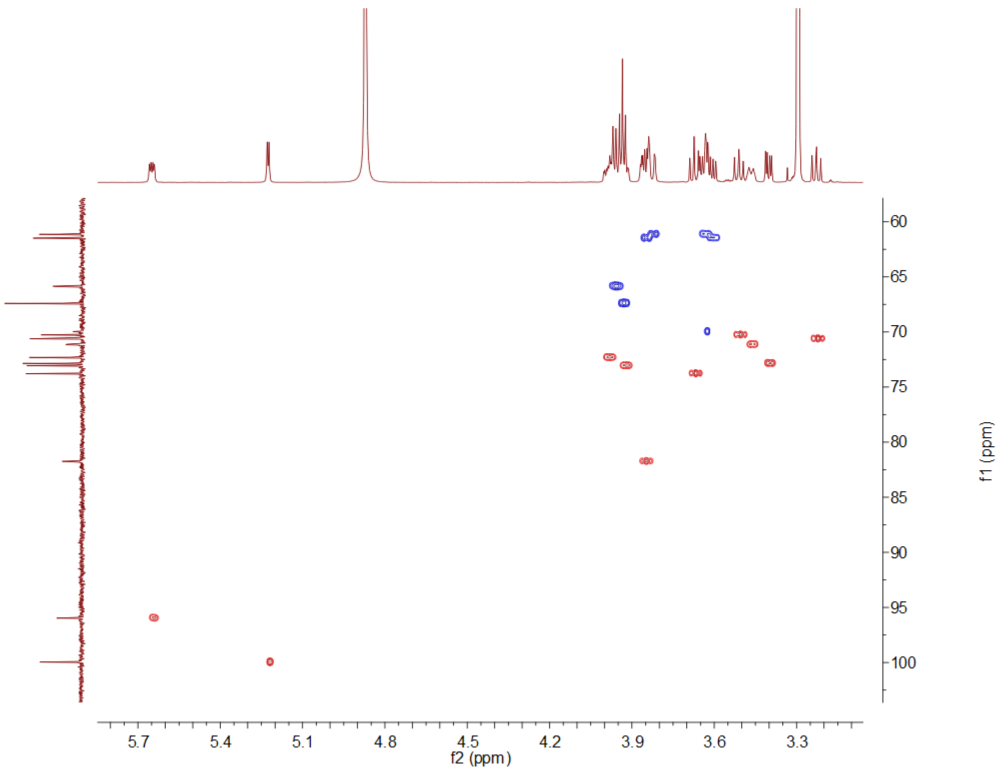

gHSQC spectrum of Glcα1,3-Glcα-PP-$(CH_2)_{11}$-OPh **3** (CD_3OD, 600/150 MHz, 25 ºC)

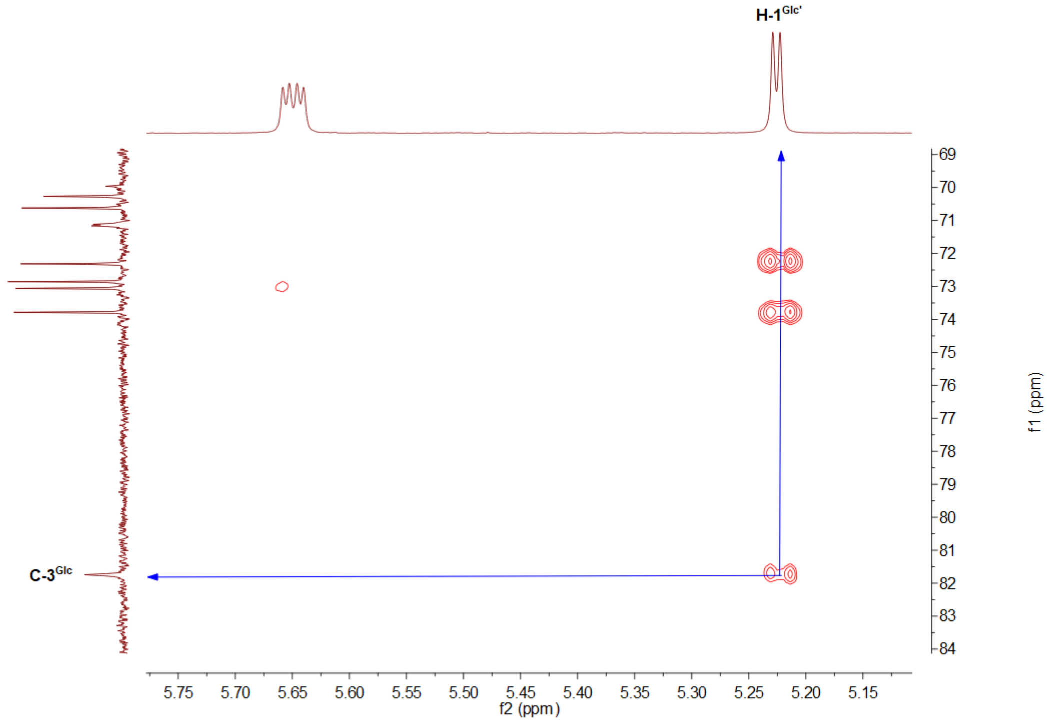

gHMBC spectrum of Glcα1,3-Glcα-PP-$(CH_2)_{11}$-OPh **3** (CD_3OD, 600/150 MHz, 25 ºC)

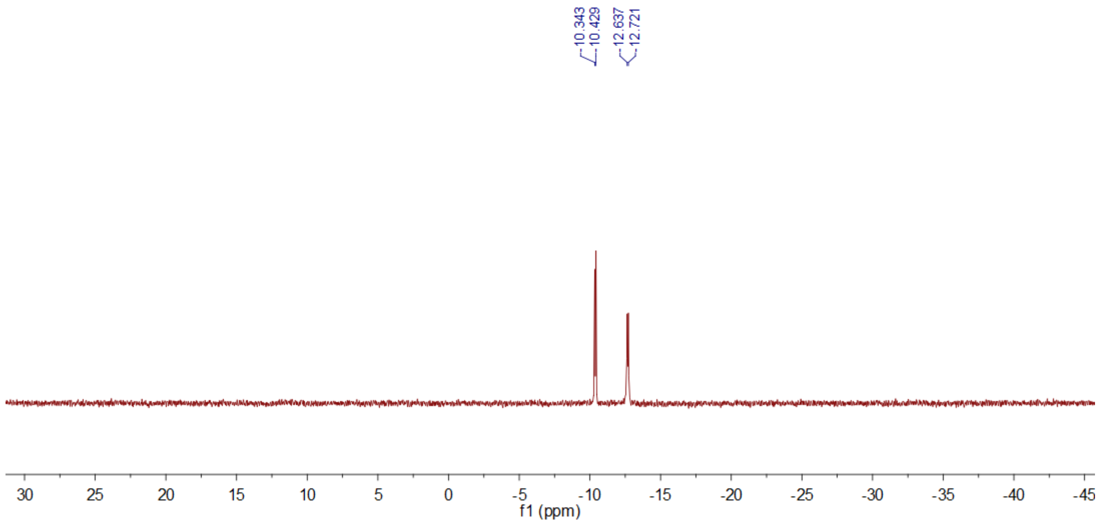

^{31}P NMR spectrum of Glcα1,3-Glcα-PP-$(CH_2)_{11}$-OPh **3** (CD_3OD, 243 MHz, 25 ºC)
